# Attentional Capture From Inside vs. Outside the Attentional Focus

**DOI:** 10.3389/fpsyg.2021.758747

**Published:** 2021-11-08

**Authors:** Greta Manini, Fabiano Botta, Elisa Martín-Arévalo, Vera Ferrari, Juan Lupiáñez

**Affiliations:** ^1^Department of Experimental Psychology, Centro de Investigación Mente, Cerebro y Comportamiento (CIMCYC), University of Granada, Granada, Spain; ^2^Department of Neuroscience, University of Parma, Parma, Italy

**Keywords:** attentional capture, perceptual load, attentional focus, distractor interference, distractor relevance

## Abstract

In this study, we jointly reported in an empirical and a theoretical way, for the first time, two main theories: Lavie’s perceptual load theory and Gaspelin et al.’s attentional dwelling hypothesis. These theories explain in different ways the modulation of the perceptual load/task difficulty over attentional capture by irrelevant distractors and lead to the observation of the opposite results with similar manipulations. We hypothesized that these opposite results may critically depend on the distractor type used by the two experimental procedures (i.e., distractors inside vs. outside the attentional focus, which could be, respectively, considered as potentially relevant vs. completely irrelevant to the main task). Across a series of experiments, we compared both theories within the same paradigm by manipulating both the perceptual load/task difficulty and the distractor type. The results were strongly consistent, suggesting that the influence of task demands on attentional capture varies as a function of the distractor type: while the interference from (relevant) distractors presented inside the attentional focus was consistently higher for high vs. low load conditions, there was no modulation by (irrelevant) distractors presented outside the attentional focus. Moreover, we critically analyzed the theoretical conceptualization of interference using both theories, disentangling important outcomes for the dwelling hypothesis. Our results provide specific insights into new aspects of attentional capture, which can critically redefine these two predominant theories.

## Introduction

Since the first studies on attention, it became clear that we cannot assimilate all the massive amounts of stimulation present in the surrounding environment but can assimilate only the intentionally focused part to achieve our current goals (Broadbent, 1958; Ruz and Lupiáñez, 2002). Thus, attention is considered a mechanism for selection (Desimone and Duncan, 1995). However, inflexible concentration on one part and fully ignoring the rest would not be adaptive in some situations (Ashinoff and Abu-Akel, 2021), and in fact, several authors have reported that some specific stimuli, such as abrupt onsets, novel stimuli (Yantis and Hillstrom, 1994), stimuli with a biological motion (Pratt et al., 2010), salient feature contrasts (Theeuwes, 1992), or emotional stimuli (Fox et al., 2002), can also attract attention even if they are not essential for the achievement of our immediate goals (Itti et al., 1998). Nevertheless, the specific mechanism underlying this function, the so-called *attentional capture*, has historically been a matter of broad debate in the attentional field (Posner, 1980; Ruz and Lupiáñez, 2002; Burnham, 2007; Theeuwes, 2010; Folk, 2015) concerning both (i) how attention voluntarily selects the relevant information and (ii) what is the destiny of task-irrelevant information.

### Locus of Selection

The first important theories approaching the above-mentioned evidence were centered on the locus of the selection, giving birth to a debate between theoretical approaches defending an *early selection* theory and those defending a *late-selection* theory. The first early selection theory was proposed by Broadbent (1958), who considered that as individual processing capacities are limited, the filtering of information should take place at early processing stages, at a sensory level, thus avoiding a cognitive overload. Furthermore, for this theory, filtering is considered as a rigid process where the selection is made based on physical task-relevant features of the incoming information, letting pass just a small amount of information to be processed (Broadbent, 1958). However, for other researchers, the selection of the relevant information is possible only after the perceptual analysis of all the stimuli (Deutsch and Deutsch, 1963). This late-selection theory states that the filter does not act on simple physical features but on stimuli perceptually fully processed, acting at the semantic level. Late-selection theories state that our perceptual capacity is unlimited and that the limitations are rather located at the response level, and therefore the selection takes place later, as a gate for information getting access to consciousness.

#### Solving the Locus of Selection

With an attempt to conciliate both perspectives, the *perceptual load theory* (Lavie and Tsal, 1994; Lavie, 1995) considers attentional capture as a function of available perceptual resources (Lavie et al., 2004). According to this theory, when the processing of the relevant information (i.e., main task) is easy and demands few resources, there would be some resources left, which would be automatically and mandatorily allocated to process the irrelevant information, aligning with late selection. However, when the relevant information is perceptually demanding, there would be no resources left and thus the irrelevant information would not be processed, implementing early selection in this case (Lavie, 2005). Thus, this perspective retakes, on one hand, the assumption from the early selection theory (Broadbent, 1958) that perception has a limited capacity; on the other hand, it recovers, from a late-selection perspective, the idea that perception is involuntary so that once the relevant information/stimuli have been selected, all resources left free from the processing of the main task will be mandatorily used to perceive the irrelevant stimuli (Lavie, 2010).

**FIGURE 1:**
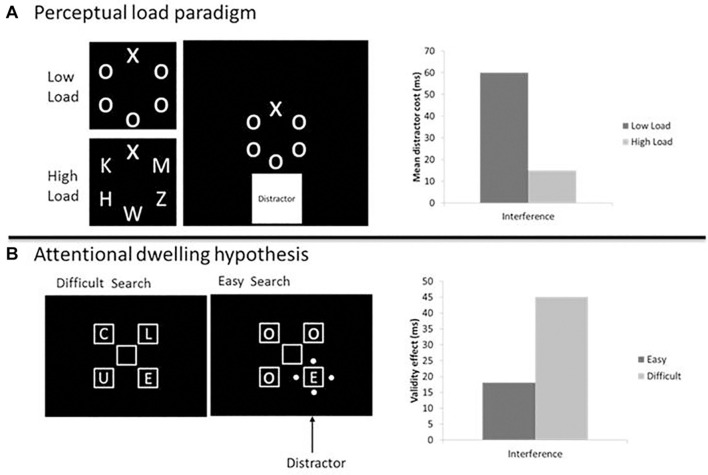
A representation of the **(A)** perceptual load paradigm by Forster and Lavie (2008b) and **(B)** attentional dwelling hypothesis by Gaspelin et al. (2016). In both cases, there was a discrimination task where participants had to report the identity of the target, choosing among two possible target letters. The load/difficulty was manipulated by increasing stimuli similarity with the target. However, in the **(A)** perceptual load paradigm, the distractor consisted in a cartoon character presented at the periphery of the relevant search array, and its interference (calculated as the difference between distractor present vs. distractor absent) decreased when the perceptual load was high. While in the **(B)** attentional dwelling hypothesis, the distractor was a stimulus onset presented in one of the potential target positions, and its interference (considered as the difference between valid vs. invalid distractor) increased in the difficult search difficulty.

The paradigm that has been typically used to test this theory comprises a visual search task, wherein participants have to discriminate a target letter (e.g., a Z vs. an M) presented among the other five non-target letters. To probe the perceptual load modulation, the target is presented either among Os (low perceptual load condition) or among heterogeneous angular letters (high perceptual load condition; see Figure 1A for such an example). To test the extent to which the irrelevant information is processed, eventually, a peripheral irrelevant distractor, usually an image or a letter (Lavie, 1995; Beck and Lavie, 2005; Forster and Lavie, 2008b), is presented in a separated location from the potential target positions. The results obtained from this paradigm have largely shown that while in the high perceptual load condition, irrelevant distractors do not elicit any interference, in the low perceptual load condition, they usually lead to a significant *interference* effect, characterized by longer response times (RTs) and/or a higher error rate when the distractor is presented compared to when it is not presented along with the target (Forster and Lavie, 2007, 2008b; Morris et al., 2020; see Murphy et al., 2016 for a full review; Santangelo et al., 2011).

This way, as stated earlier, the perceptual load theory solves the debate about the early vs. late selection, thus explaining the fate of the completely irrelevant information outside the focus of attention (i.e., the stimuli presented at the periphery of the attended circular array).

### Attentional Capture Debate

In addition to the early/late-selection debate, other theories arose taking into account new aspects concerning attentional capture. Specifically, these theories tried to analyze how we process the relevant stimuli after their initial selection. That is, once we select the task-relevant information (i.e., letters within a circular array), how do we select the target and the correct response among all potentially relevant objects/responses? How does attention guide the competition among the potentially relevant stimuli (Desimone and Duncan, 1995), and why and how do some of these stimuli capture attention more than others in this competition?

These questions were not well taken into account by the previously mentioned approaches, generating another important debate in the attentional capture literature (Luck et al., 2021) about whether a selection within the attended information is mainly or exclusively guided by bottom-up or top-down processes. On one hand, the *stimulus-driven* theory (Yantis and Jonides, 1984; Theeuwes, 1991, 1994, 2004; Yantis and Hillstrom, 1994) conceives attentional capture as directly driven by stimulus salience, and thus under the control of bottom-up processes. On the other hand, alternative *goal-driven* perspectives, such as the *contingent capture theory* (Shibuya, 1993; Folk et al., 1994; Folk and Remington, 1998), consider the attentional capture contingent on attentional sets as the result of top-down processes.

In particular, the *stimulus-driven* perspective is sustained by the results based on the additional singleton paradigm (Theeuwes, 1991, 1992), a search task in which two stimuli (the target and the distractor within the same relevant search array), which are singletons in different dimensions, are (sometimes) simultaneously presented. For example, the target is defined as a shape singleton, i.e., the only different shape stimuli among other same-shape stimuli, and a distracting color singleton is presented in some of the trials, i.e., one of the same-shape distractors is presented in a different contrasting color. Attentional capture in this context is measured as distractor interference, an increment in RTs on trials with both the target and the distractor in comparison with target-alone trials. The singleton distractor, highly salient but task-irrelevant, is believed to automatically attract attention, guided by the bottom-up processes that exclusively depend on the relative salience of the attention-capturing object against the other stimuli within the attended search display (Theeuwes, 1992, 1994). Whenever the saliency of the irrelevant singleton is reduced, no cost is observed, indicating the existence of pre-attentive analyses in the early stages of selection processes, which allow the shift of attention to the location of the most salient feature (Theeuwes, 2010).

On the contrary, goal-driven theories of attentional capture, such as the contingent capture hypothesis (Folk et al., 1992), rather consider that attentional capture is fully dependent on *goal-driven* attentional sets. Using typical paradigms to gather evidence supporting this theory, participants have to search for and respond to the target that is presented in one of several possible target positions. The target presentation is preceded by a distracting non-predictive cue in one of the possible target locations (in line with the additional singleton paradigm). Note that in this case attentional capture is measured as the difference in RTs between valid trials, i.e., the target appears in the same position as the cue does, and invalid trails, i.e., the target appears in a different position from the cue, resulting in what is called a *cue validity effect*. This paradigm typically shows that only salient cues sharing features with the target (i.e., task-relevant conditions) do in fact capture attention and produce interference, whereas they have no effect when they do not share any feature with the target (e.g., an irrelevant red salient distractor will only capture attention when we are searching for a relevant red target) (Folk et al., 1994; Folk and Remington, 1998). Even if, recently, the same authors have supported the idea that infrequent abrupt onsets are immune to the contingent capture (Folk and Remington, 2015), which seems to be dependent on the task set (Schönhammer and Kerzel, 2018). These findings are commonly used to support the idea that the participant’s goals, which create top-down control settings, have a major influence on attentional capture, modifying bottom-up signals (Folk et al., 1992; Burnham, 2007; Folk, 2013).

#### The Attentional Dwelling Hypothesis: A Possible Solution to the Attentional Capture Debate

To solve this controversy, Gaspelin et al. (2016) and Ruthruff et al. (2020) proposed a solution that mirrors that of perceptual load for the early late debate: the *attentional dwelling hypothesis.* According to this theory, distracting information always captures attention, but its manifestation on the observable behavior primarily depends on the visual search difficulty (which resembles perceptual load), with attentional capture by distracting stimuli producing a stronger effect the more difficult is to discriminate the target. When the visual search task is easy, the presence of a salient distractor would have little influence on performance. However, with more difficult tasks (similar to high load conditions in Lavie’s theory), the time needed to find and respond to the target would open up a window for interference, thus salient distractors have a larger influence on performance. To test this hypothesis, Gaspelin et al. (2016) manipulated the visual search difficulty and measured the effect of an attention-capturing distractor that could appear in one of the potential target positions (see Figure 1B), either the target (i.e., valid) or one of the non-target (i.e., invalid) positions. The results showed that the validity effect (i.e., the difference between invalid and valid conditions) was indeed modulated by the task difficulty in the predicted direction: it was larger for the difficult search task than for the easy search task.

These findings support the hypothesis that the attentional interference of the distractor depends on the search task difficulty, with higher search task difficulty leading to greater interference. It is important to note, however, that in this case the manipulation of the perceptual load, or the task difficulty, modulates attentional capture in an opposite way to the results typically observed in Lavie’s perceptual load paradigm and theory.

### Inside vs. Outside the Attentional Focus

Considering the two aforementioned perspectives, perceptual load, and attentional dwelling, even if the starting point and the theoretical framework of both theories are different, they both point to the perceptual load or search difficulty as a critical factor. However, both approaches present a strong discrepancy about the mechanisms underlying the modulation over attentional capture and set opposite predictions regarding the effect of the perceptual load/search difficulty over the processing of distractors. Indeed, both theories are strongly supported by the opposite patterns of data, each predicted by its corresponding different view. While Lavie et al. (Beck and Lavie, 2005; Cartwright-Finch and Lavie, 2007; Forster and Lavie, 2008a) typically observe much reduced interference on tasks with high perceptual load (i.e., difficult search), Gaspelin et al. (2016) and Ruthruff et al. (2020), on the contrary, show stronger interference with a difficult search. Note that, despite these opposite results, however, in both paradigms, the stimuli and especially how the perceptual load/search difficulty is manipulated are quite similar. Furthermore, this evidence could indicate that, despite the different cognitive processes underlying each theory, the task difficulty manipulation could be fundamental not only for comparing but also for an understanding of these processes. Surprisingly, however, to the best of our knowledge, both perspectives have not been jointly investigated systematically, and in fact, the published studies from the search difficulty approach rarely cite those of the perceptual load approach and *vice versa*.

Thus, based on the aforementioned evidence, this study aimed at jointly testing and comparing the perceptual load theory (Lavie, 1995; Lavie et al., 2004) and the attentional dwelling hypothesis (Gaspelin et al., 2016; Ruthruff et al., 2020) within the same experimental paradigm, to further disentangle the critical variables underlying these opposite results. To do this, we manipulated the distractor position so that it could be either entirely *irrelevant* (Forster, 2013; outside the test array; to test the perceptual load theory) or potentially *relevant* (inside the search array; to test the attentional dwelling hypothesis). Furthermore, presenting both distractors within the same paradigm, thereby using the same load manipulation, allows us to determine whether the opposite effects of the perceptual load on attentional capture merely depend on the differences between paradigms or rather need a more conceptual explanation.

As derived from the abovementioned introduction to the topic, we had the hypothesis that the two different distractors (i.e., potentially relevant and entirely irrelevant) are processed differently, eliciting different types of interference. We suggest that coping up with the interference elicited by each distractor type places different attentional demands, which might explain the opposite interaction with the perceptual load, perhaps reflecting the different mechanisms taking place to deal with the two types of distractors. Therefore, we predicted that (i) while the entirely irrelevant distractor would elicit larger interference under low compared to high perceptual load, (ii) the interference pattern of the potentially relevant distractor would be the opposite, namely that the greatest interference would be observed under high perceptual load. If the results of this study differ from our predictions, we could assume that the opposite outcomes are simply caused by the use of different paradigms. On the contrary, if the results are in line with the aforementioned predictions, we could consider other theoretical explanations such as the involvement of different mechanisms for dealing with the interference from inside vs. outside of the attentional focus.

Here, we present the first experiment where we tested our main hypotheses by combining both distractors in the same paradigm and manipulating the perceptual load/task difficulty as mentioned earlier. For the irrelevant distractor, even if the typical results supporting the perceptual load theory are found by using either letters’ or images’ distractors, normally cartoon characters (Forster and Lavie, 2008b), we decided to use fully irrelevant distractors (Forster, 2013) that have been found to create interference even when presented peripherally to the relevant target search array (Martín-Arévalo et al., 2015). For the potentially relevant distractor manipulation, we presented a letter of the search array in red, whereas others were uniformly presented in black (Theeuwes, 1994; Ruthruff et al., 2020). It is important to note that the use of this color singleton letter differs from both the additional singleton paradigm (Theeuwes, 1991, 1992) and the contingent capture paradigm (Folk et al., 1992). Compared to the former, the colored letter could be also the target and compared to the latter, the percentage with which the distractor occurs is higher than in a typical paradigm (see Becker, 2007). However, this manipulation of the color dimension allows us to manage the relevance of distractors without using an abrupt onset (Gaspelin et al., 2016), a stimulus that is considered by some authors as a *special* distractor-type immune to suppression (Folk and Remington, 2015; Ruthruff et al., 2019; however, see Schönhammer and Kerzel, 2018).

Based on this first experiment, we developed three experimental series, with two experiments each, in which, besides testing the same hypotheses, we further investigated the other variables that could be acting on the observed results. In the first series, we slightly modified our original paradigm to detect to what extent variables as the stimuli time exposure (Experiment 2a) or the mental set (Experiment 2b) could modulate the results. Particularly, in Experiment 2a, we reduced the stimuli time exposure that represents an important aspect to enhance the interference by distractors, especially the irrelevant ones (Forster and Lavie, 2008b). In Experiment 2b, to check for the possible mental-set modulations, the two distractors (i.e., relevant and irrelevant) were presented in a separate block (Theeuwes et al., 2004; Belopolsky and Theeuwes, 2010; Biggs and Gibson, 2018). In the second series (Experiments 3a and 3b), we changed the irrelevant distractor to a cartoon character (similar to Forster and Lavie, 2008b, a; Forster et al., 2014). We reasoned that the distractor features would enhance its saliency and therefore its interference, potentially interacting more strongly with the load/task difficulty. Finally, in the third series of experiments (Experiments 4a and 4b), we further changed the outside-the-focus distractor to a letter to analyze whether sharing features with the target could modulate the observed results (Duncan and Humphreys, 1989; Wolfe, 1994; Bichot and Schall, 1999). In this case, both relevant and irrelevant distractors were letters (Table 1 shows the different manipulations made for each experimental series).

**TABLE 1:** Mean response time (RT; in ms), SD RT, and error rates for each condition.

Experiment	Irrelevant distractor	Distractor presentation	Load	Absent		Peripheral		Invalid		Valid	
				RT	Error	RT	Error	RT	Error	RT	Error
1	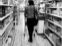	Until response (1200 ms)	Low	**503.7** 55.91	4.69%	**519.4** 60.55	4.81%	**537.5** 86.62	5.62%	**496.7** 60.31	3.86%
		Within blocks	High	**675** 73.53	4.74%	**691.6** 70.83	4.74%	**747.6 103.9**	7.88%	**589.3** 82.19	3.24%
2a	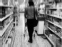	200 ms	Low	**489.6** 55.36	5.88%	**493.3** 57.14	4.62%	**520 65.42**	6.37%	**483.2** 53.83	3.45%
		Within blocks	High	**635.2** 63.97	10.82%	**647.8** 72.32	10.76%	**661.8** 69.81	17.52%	**573.3** 83.74	8.97%
2b	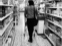	200 ms	Low	**530.5** 98.28	5.56%	**550.8** 121.49	6.23%	**623.7** 138.24	8.50%	**535.9** 116.55	4.51%
		Between blocks	High	**674.7** 114.43	10.98%	**691** 130.49	12.49%	**749.6** 162.75	12.77%	**589.96** 121.8	7.02%
3a	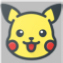	200 ms	Low	**486.2** 61.05	6.62%	**495.4** 65.79	7.34%	**524.5** 76.48	6.76%	**483.2** 65.86	8.07%
		Within blocks	High	**628.3** 79.63	11.14%	**650.3** 93.12	12.64%	**671.4** 97.44	14.44%	**579.2** 81.75	8.83%
3b	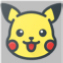	200 ms	Low	**489.1** 35.99	6.49%	**501.6** 44.55	5.06%	**549.7** 67.32	7.98%	**490.4** 43.26	5.06%
		Between blocks	High	**643.3** 54.76	10.93%	**653.6** 75.86	14.73%	**719.2** 97.21	16.24%	**563.1** 57.04	4.19%
4a	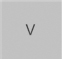	200 ms	Low	**500.8** 52.56	5.0%	**520.2** 63.64	3.39%	**530.6** 69.24	6.91%	**496.7** 60.97	4.06%
		Within blocks Black letter	High	**635.2** 63.66	10.46%	**641.2** 72.12	11.33%	**688.3** 69.58	12.53%	**586** 61.74	6.78%
4b	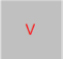	200 ms	Low	**509.4** 58.86	5.22%	**521.5** 60.65	4.64%	**538.5** 59.41	8.16%	**500.7** 56.21	3.66%
		Within blocks Red letter	High	**652.9** 64.9	11.86%	**658.4** 87.55	12.51%	**703.2** 88.38	14.63%	**588.3** 59.71	5.34%

*In order to avoid copyright violation, the image shown in Experiment 3a and 3b is not a real stimulus used in the experiments (image from Flaticon.com). Mean RT values are presented in bold, in order to distinguish it from SD RT.*

In summary, in this series of experiments, we aimed at jointly, empirically, and theoretically, exploring not only whether and how the perceptual load/search difficulty differently affected distractors’ processing depending on their relevance, but also which variables might be regulating this potential interaction.

## Experiment 1

**FIGURE 2:**
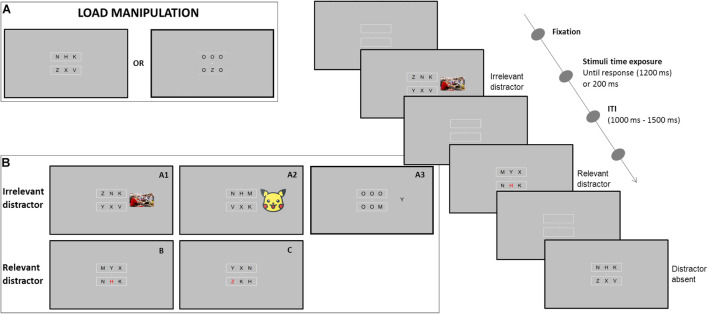
Representation of the paradigm of the first experimental series. The upper part shows **(A)** the manipulation of the load/difficulty of the task, while in the lower part **(B)** are shown all the possible distractors. Depending on the experiment, the irrelevant distractor could be a peripheral **(A1)** image, **(A2)** cartoon character, or a **(A3)** letter (black or red depending on the experiment). While the relevant distractor consisted in presenting one of the letters colored in red, half of the time it was **(B)** a non-target letter, the other half **(C)** the red letter coincided with the target. The two types of distractors (relevant and irrelevant) were presented in the same block or in separate blocks depending on the experiment. On the right side, the temporal presentation of the paradigm is represented.

Here, we used a discrimination task with the load/task difficulty manipulation similar to the one used in Forster and Lavie (2008b), where the load was determined by the similarity of non-target letters, and the distractor manipulation by either presenting an image peripherally to the relevant search array (the fully irrelevant distractor), to test the perceptual load theory, or a non-target letter in red (a relevant distractor) in the search target array, to test the dwelling hypothesis (see Figure 2). Both the variables (load and distractor) were manipulated within the same block. We hypothesized that the perceptual load would affect the distractors’ interference differently depending on their relevance. More specifically, we expected (i) the interference from irrelevant distractors to be lower in high compared to low perceptual load conditions, as predicted by the perceptual load theory (Lavie et al., 2004) and (ii) the interference from potentially relevant distractors to be higher in high compared to low perceptual load conditions, as proposed by the dwelling hypothesis (Gaspelin et al., 2016).

### Methods

#### Participants

To calculate the needed sample size, we used the G^∗^Power 3.1.9.4 (Faul et al., 2009) to perform a power analysis based on Forster and Lavie (2008b) study. We took into account the effect size of the interaction load × irrelevant distractor observed in Experiment 2b, *F*(1,15) = 5.82, *p* = 0.029, η*_*p*_*^2^ = 0.28, in which the two different distractors are also used. To observe the interaction with an alpha of 0.05 and a power of 0.80, the total number of participants needed was 23. Therefore, for this first experiment, a total of 25 healthy volunteers participated (1 left-handed; 20 women; mean age of 22.24 years, SD = 3.08). In this and all the following experiments, participants were naïve students from the University of Granada, who signed informed consent, and reported normal or corrected to normal vision. All the experiments were conducted as per the ethical guidelines laid down by the University of Granada, in accordance with the ethical standards of the 1964 Declaration of Helsinki, as part of a larger research project approved by the University of Granada Ethical Committee (175/CEIH/2017).

#### Apparatus and Stimuli

The experiment was programmed with E-prime 2 software (Schneider et al., 2002). The stimuli were presented on a 21-inch computer screen, at an approximate viewing distance of 58 cm. All stimuli were drawn in white against a gray background. Each display contained the fixation point (0.5° × 0.5°) and two rectangular boxes below and above the fixation point (see Figure 2). Each box subtended 3.9° in height ×9.3° in width and all of them were positioned 1° away from the central fixation point. Each box always contained three letters that, depending on the condition, could be presented all in black color or two in black and one in red (i.e., the distractor from inside the attentional focus). Furthermore, a picture of the natural scene (6.7° in width ×5.7° in height) served as the peripheral distractor (i.e., the distractor from outside the attentional focus) presented in the left or right visual field (at a distance of 5.5° from the fixation point). A total of 11 pictures were selected from the International Affective Picture System (IAPS; Lang et al., 2005) and depicted people in neutral contexts (5.25 = valence ratings, 3.54 = arousal ratings).

#### Procedure

The sequence of events in each trial is illustrated in Figure 2. The fixation point and the two boxes remained fixed on the screen. Participants were required to keep their eyes on the fixation point throughout all the trials. Then, the target was presented within the relevant search array alone or simultaneously with distractors, until response or for a maximum of 1,200 ms, and the responses were registered up to 1,200 ms. The inter-trial interval (ITI) was randomized between 1,000 and 1,500 ms to avoid expectations. Furthermore, to reproduce different perceptual load conditions, the target (Z or M) was presented among the other five letters: all Os in the low load condition, and all different angular letters in the high load condition. In the latter case, non-target letters were selected based on target similarity (i.e., W, X, Y, V, H, and N). These two load conditions were mixed within the same block, each one representing half of the trials. The target position was randomized between all the six possible spatial positions. Participants were instructed to discriminate the identity of the letter by pressing an equivalent “Z” or “M” on the keyboard whenever one of the two targets was on the screen. Thus, to test the perceptual load theory (Lavie, 2010) in 10% of the trials, the irrelevant/outside-the-focus distractor (a peripheral neutral image^1^) was presented at the periphery of the relevant search array. To reproduce the validity effect pattern predicted by the dwelling hypothesis (Gaspelin et al., 2016), another 20% of the trials presented one of the six letters in red, and in half of these trials (i.e., 10%) the target was presented in red (valid condition), and in the other half (another 10%), a non-target letter (distractor) was presented in red (invalid condition). By doing so, the presence of a red letter becomes a relevant/inside-the-focus distractor due to its potentiality of being the target. The remaining 70% of the trials presented the target without any distractor (absent condition).

#### Design

The experiment comprises a three-factor design, with all variables manipulated within participants. All the factors had two levels: the perceptual load (low vs. high), irrelevant distractor (absent vs. peripheral condition), and relevant distractor (invalid, a non-target red letter, vs. valid condition, a target red letter). The experiment was comprised of 20 practice trials, which were not further analyzed, followed by 6 blocks of 80 experimental trials, of which 70% of them belonged to the condition without any distractor, and 10% to any of the other three conditions (half of them with low load and another half with high load).

For the analysis of the results, to have a comparison with both the original paradigms that we have considered in this experiment, we calculated the interference of each distractor type as follows: on one hand, we considered the interference of the irrelevant distractor as the difference between its presence (peripheral condition) and its absence (absent condition), exactly as contemplated in the perceptual load theory (Forster and Lavie, 2008b). On the other hand, as in the attentional dwelling hypothesis (Gaspelin et al., 2016), the interference of the relevant distractor was measured as a validity effect, by subtracting the valid condition (red target) from the invalid condition (a red non-target letter). Thereby, we calculated two 2 × 2 ANOVAs, one for each distractor type, to study their interaction with the perceptual load. For the irrelevant distractor, we performed an ANOVA between the perceptual load (low vs. high) and irrelevant interference (absent vs. peripheral condition), while for the relevant distractor, we performed another ANOVA between the perceptual load and relevant interference (valid vs. invalid condition).

### Results

Mean RTs and error rates for each experimental condition are presented in Table 1, whereas the distractor effects are presented in Table 2. Trials in which no responses were recorded or trials in which an incorrect response was made were excluded from the RT analysis (8.45% of the trials). In addition, correct response trials faster than 200 ms were considered anticipations and were also excluded from the RT analysis (0.01% of the trials).

**TABLE 2:** Interference effect (in ms) for each distractor type, load, and experiment.

		Experiment						

		1	2a	2b	3a	3b	4a	4b
		
	Load							
Irrelevant interference Peripheral – Absent	Low	16	4	20	9	13	20	12
	High	17	12	16	22	10	6	6
Relevant interference Invalid – Valid	Low	41	37	88	41	59	34	38
	High	158	89	151	92	156	102	115

As mentioned earlier, two 2 × 2 ANOVAs were conducted for both mean RT and error rates, one for the irrelevant distractor, to test the perceptual load theory, and the other for the relevant distractor, to test the attentional dwelling hypothesis.

#### Irrelevant Distractors

**FIGURE 3:**
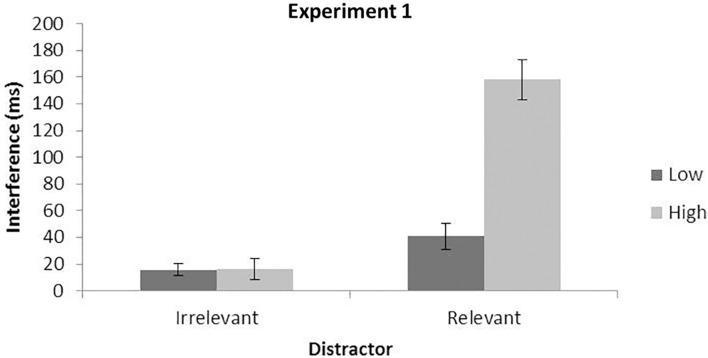
Mean interference effect for irrelevant (distractor present — absent) and relevant (invalid — valid) distractors as a function of perceptual load. Data from Experiment 1. Error bars represent standard error of the mean.

Response time analysis showed a main effect of the perceptual load and the distractor, *F*(1,24) = 465.98, *p* < 0.0001, η*_*p*_*^2^ = 0.95, and *F*(1,24) = 15.61, *p* = 0.0006, η*_*p*_*^2^ = 0.39. Participants were faster in the low load than in the high load conditions and in the distractor absent than in distractor present trials. Importantly, however, no interaction between the two factors was observed, *F*(1,24) = 0.01,*p* = 0.9319, η*_*p*_*^2^ < 0.01. In particular, as shown in Table 2 and Figure 3 (left part), a similar interference effect was observed for both the low (distractor present–absent conditions; 16 ms) and high (17 ms) perceptual load conditions. It is important to note that the main effect of the distractor is significant despite the production of a small interference effect. Error rate analysis did not show any significant effect neither for the load nor for the distractor or the interaction between the two factors, with all *Fs* < 1.

#### Relevant Distractors

The RT analysis in trials with relevant distractors showed, apart from the two main effects of the perceptual load and the distractor, *F*(1,24) = 220.79, *p* < 0.0001, η*_*p*_*^2^ = 0.90 and *F*(1,24) = 139.48, *p* < 0.0001, η*_*p*_*^2^ = 0.85, respectively, a significant interaction, *F*(1,24) = 37.56, *p* < 0.0001, η*_*p*_*^2^ = 0.61, with larger interference in the high (invalid–valid conditions; 158 ms) than in the low (41 ms) perceptual load condition, as can be observed in Figure 2 (right side). The error rate analysis showed a significant main effect of the distractor, *F*(1,24) = 14.04, *p* < 0.0010, η*_*p*_*^2^ = 0.37, with more errors for the invalid than for the valid trial. A main effect of the load and an interaction failed to reach significance, both *ps* > 0.130.

### Discussion

Partially in line with previous literature studies, the present data show that the presence of the distractor (either relevant or irrelevant) interfered with the search of the target. Moreover, we also observed larger RTs for high than for low load conditions in general. Interestingly, the effect of interference interacted differently with the perceptual load depending on the relevance of the distractor, thus pointing to the distractor relevance as a critical factor for the modulation of attentional capture over interference. More specifically, the interference of the potentially relevant distractor increased significantly in the high compared to the low load condition, as expected (Gaspelin et al., 2016; Ruthruff et al., 2020). Furthermore, as predicted, this pattern was not observed for the interference from an entirely irrelevant distractor. However, contrary to our more specific predictions based on the perceptual load hypothesis (Lavie, 1995; Forster and Lavie, 2008a), the interference by the irrelevant distractor rather than being weaker in the high compared to the low load condition did not show any significant interaction with the load factor.

We reasoned that this unexpected discrepancy between the present findings and the typical Lavie’s results could have (at least) two possible explanations: (i) the exposure time of the stimuli and/or (ii) the fact that the different distractors (relevant and irrelevant) were presented mixed within the same block. Regarding the first point, all the stimuli (target and distractors, when presented) were displayed until response or 1,200 ms in this study. Based on a few previous literature studies, we hypothesized that a shorter stimulus exposure time might emphasize the pressure for target detection, thus leading to a larger interference effect by peripheral distractors (Yeshurun and Marciano, 2013). Indeed, in the original study of Forster and Lavie (2008b), they observed no modulation of the load manipulation with a long exposure time of stimuli (as given here), concluding that it could also involve eye movements, with RTs reflecting eye movements rather than a strictly attentional effect (Forster and Lavie, 2008b), an aspect that can be solved by reducing the stimuli time exposure. Concerning the second point, at the same time, it is also possible that the presence of both distractors (relevant and irrelevant), within the same block of trials, could probably lead participants to adopt a different attentional set to the one induced by a typical perceptual load paradigm (Theeuwes et al., 2004). Indeed, even if we observed a significant interference by the irrelevant distractor, this was smaller (16 ms) as compared to the interference normally observed in perceptual load paradigms [∼51 ms in Forster and Lavie (2008b); ∼35 in Lleras et al. (2017); and ∼28 ms in Morris et al. (2020)]. As demonstrated by Belopolsky et al. (2007), attentional capture seems to be modulated by the size of the attentional window; namely, the same distractor stimulus diminishes its capacity to interfere with the task when it falls outside the attentional window. In this case, the presence of the relevant distractor could induce participants to adopt a narrow attentional focus, preventing the interference of the irrelevant distractor. Specifically, the fact that the relevant distractor is presented within the relevant search array, requires the first target/distractor discrimination to complete the task, which could be causing participants to narrow the attentional focus to the central spatial area where the relevant search array is located. As a result, because the irrelevant distractor is presented peripherally to the relevant search array, it would fall outside the attentional window, causing a reduction of the interference to the task (Belopolsky and Theeuwes, 2010; Biggs and Gibson, 2018). All these considerations led to the next experimental series.

## Experiment 2

The same task as shown in Experiment 1 was used here except for the following: stimuli duration was limited to 200 ms, and the distractor presentation could be either within or between the blocks. In both Experiments 2a and 2b, stimuli duration was limited to 200 ms, but in Experiment 2a, both the distractor conditions (relevant and irrelevant) were presented mixed within blocks, while in Experiment 2b they were presented in different blocks. We carried out this experimental series to (i) replicate the main findings observed in Experiment 1, while (ii) putatively increasing the interference effect potentially generated by peripheral distractors, and (iii) controlling the potential influence of the attentional set elicited by the distractor presentation.

### Methods

#### Participants

Two new samples of participants took part in the present experimental series: Experiment 2a, with a total of 25 healthy volunteers (all right-handed; 17 women; mean age of 24 years, SD = 3.15), with 1 participant excluded from the analysis because of a low accuracy rate (49%), and Experiment 2b, with a total sample of 24 participants (2 left-handed; 20 women; mean age of 21.67 years, SD = 3.02).

#### Procedure and Design

Stimuli and procedure were identical to those of Experiment 1, with the exception that (i) in both experiments the stimuli time presentation was fixed at 200 ms, and additionally, in (ii) Experiment 2b, the two distractor types (relevant and irrelevant) were presented separately between blocks. Specifically, the first block, divided into six subblocks of 64 samples each, presented only the peripheral distractor (10% of the trials) and the absent condition, while the second block, divided into only two subblocks of 48 samples each, displayed only the relevant distractors: valid and invalid conditions (presented on 50% of these blocks’ trials). As shown in Experiment 1, both experiments consisted of an identical three-factor design, with all variables being manipulated within participants. Also, in this case, we performed two 2 × 2 ANOVAs for both mean RT and error rates, one for the irrelevant distractor, to test the perceptual load theory, and the other for the relevant distractor, to test the attentional dwelling hypothesis.

### Results

#### Experiment 2a

Mean RT and error rates for each experimental condition are shown in Table 1. Incorrect trials were excluded from the RT analysis (11.77% of the trials), and the trials with correct responses but faster than 200 ms were considered anticipations (0.03% of the trials).

##### Relevant Distractors

**FIGURE 4:**
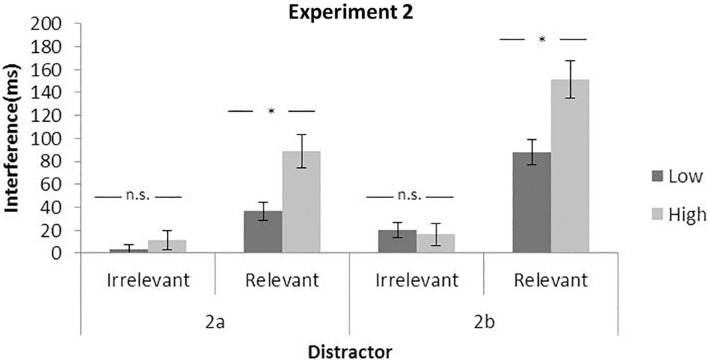
Mean interference effect for irrelevant (distractor present — absent) and relevant (invalid — valid) distractors as a function of perceptual load. Data from Experiment 2. Error bars represent standard error of the mean.

The 2 × 2 ANOVA for RTs showed a main effect of the load, *F*(1,23) = 252.18, *p* < 0.0001, η*_*p*_*^2^ = 0.92, but not of the distractor, *F*(1,23) = 3.59, *p* = 0.0709, η*_*p*_*^2^ = 0.13. The interaction between the two factors also failed to reach significance, *F*(1,23) = 0.61, *p* = 0.4431, η*_*p*_*^2^ = 0.03. As can be observed in Figure 4 (left part), the interference effect was not significantly different for the two perceptual load conditions (4 and 12 ms, for low and high perceptual load, respectively).

Similar to the first experiment, the error rates did not show a significant effect of neither the distractor condition nor its interaction with the load, both *Fs* < 1. However, a main effect of the perceptual load was significant, *F*(1,23) = 20.28, *p* = 0.0002, η*_*p*_*^2^ = 0.47, with higher error rates in high than low perceptual load (see Table 1).

##### Relevant Distractors

In contrast, the relevant distractor showed a main effect of the two factors, *F*(1,23) = 122.83, *p* < 0.0001, η*_*p*_*^2^ = 0.84 and *F*(1,24) = 45.65, *p* < 0.0001, η*_*p*_*^2^ = 0.67. Moreover, there was also clearly a significant interaction, *F*(1,23) = 13.09, *p* = 0.0014, η*_*p*_*^2^ = 0.36, with larger interference with high (89 ms) than low (37 ms) perceptual load (see Figure 4).

In this case, the error rates showed that the two main effects were significant, respectively, *F*(1,23) = 40.34, *p* < 0.0001, η*_*p*_*^2^ = 0.64 and *F*(1,23) = 13.73, *p* = 0.0012, η*_*p*_*^2^ = 0.37, as well as showed the interaction between them, *F*(1,23) = 4.71, *p* = 0.0406, η*_*p*_*^2^ = 0.17. As it can be observed in Table 1, the error rates incremented in high perceptual load, and participants committed the highest errors when the relevant distractor was presented.

#### Experiment 2b

Incorrect trials (13.97% of the trials) and anticipations (0.07% of the trials) were excluded from the RT analysis.

##### Irrelevant distractor

In this case of RTs, the results showed a main effect of both the perceptual load, *F*(1,23) = 363.22, *p* < 0.0001, η*_*p*_*^2^ = 0.94, and the distractor, *F*(1,23) = 7.78, *p* = 0.0105, η*_*p*_*^2^ = 0.25, but again the interaction between them failed to reach significance, *F*(1,23) = 0.14, *p* = 0.7114, η*_*p*_*^2^ = 0.01. As shown in Figure 4, the interference of the irrelevant distractor is similar in both low (20 ms) and high load (16 ms) conditions.

Error rates presented a significant main effect only of the load, *F*(1,23) = 15.92, *p* = 0.0006, η*_*p*_*^2^ = 0.41, with more errors in the high perceptual load condition. The main effect of the distractor and its interaction with the load was not significant, both *ps* > 0.2

##### Relevant distractor

Response time results showed not only the two main effects of the load and distractor, *F*(1,23) = 119.65, *p* < 0.0001, η*_*p*_*^2^ = 0.84 and *F*(1,23) = 103.63, *p* < 0.0001, η*_*p*_*^2^ = 0.82, but also a significant interaction between them, *F*(1,23) = 17.58, *p* = 0.0003, η*_*p*_*^2^ = 0.43. Figure 4 shows the interference of the relevant distractor, which is larger in high (151 ms) than in low (88 ms) perceptual load conditions.

Error rates presented only the significant main effects of the load and distractor, *F*(1,23) = 5.17 *p* = 0.0326, η*_*p*_*^2^ = 0.18 and *F*(1,23) = 11.75, *p* = 0.0023, η*_*p*_*^2^ = 0.34.

### Discussion

In line with Experiment 1, the present data show that (i) the presence of the distractor interfered with the task even if the interference of the irrelevant distractor failed to reach significance in Experiment 2a, and (ii) both RTs and error rates increased in the high load condition. In addition, (iii) in both experiments, load modulated the interference quite differently for relevant and irrelevant distractors. On one hand, the perceptual load interacted significantly with relevant distractors, with larger interference in the high load compared to the low load condition, confirming the attentional dwelling hypothesis. However, once again, no significant interaction was observed with the irrelevant distractor. Again, these results are in contrast with the perceptual load theory and seem to indicate that both the stimuli time exposure and distractor presentation (within or between blocks) are not modulating this discrepant pattern of results.

Despite the consistency between experiments, a possible explanation for the lack of replication of the typical perceptual load results may depend on the nature of the peripheral distractors used up to now. More specifically, even if many previous studies observed a decrement of the interference in high load using completely irrelevant distractors (Beck and Lavie, 2005; Forster and Lavie, 2007, 2008a,b, 2016; Morris et al., 2020), it is possible that the irrelevant distractor used until now was not salient enough to create the interference sufficiently strong—as suggested by Experiment 2a where a main effect of the irrelevant distractor failed to reach significance.

To further analyze an interplay between the perceptual load and the distractor relevance, in the next experimental series we tested whether the increased saliency of the irrelevant distractor could favor the modulation of the interference by the perceptual load. To do so, in Experiment 3, the irrelevant distractor was replaced by a simpler and more salient stimulus, namely famous cartoon characters previously used in perceptual load theory tasks (Forster and Lavie, 2008a), to produce the predicted modulation on the perceptual load over the irrelevant distractor interference.

## Experiment 3

This experimental series was identical to Experiment 2 except that the peripheral distractor was replaced by cartoon characters similar to the ones used in Forster and Lavie (2008b), to increase the distractor interference.

As shown in a previous experiment, we divided the participants into two equal groups: in the first group (Experiment 3a) both types of distractors, relevant and irrelevant were presented within blocks, while participants of the second group (Experiment 3b) carried out a task with the two types of distractors separated in different blocks.

### Methods

#### Participants

One new sample, with a total of 48 healthy volunteers, participated in this experimental series, with participants being randomly assigned to one of the two possible conditions. About 24 subjects participated in Experiment 3a (4 left-handed; 18 women; mean age of 20.79 years, SD = 2.23), and the other 24 participants were assigned to Experiment 3b (1 left-handed; 14 women; mean age of 21.96 years, SD = 2.27). One participant was eliminated (Experiment 3b) due to problems during data collection.

#### Procedure and Design

The procedure was performed similar to Experiment 2, except for the peripheral distractor used: the four images of cartoon characters (Pikachu, Doraemon, Mickey Mouse, and SpongeBob). Again, the experiment consisted of a three-factor design, with all variables manipulated within participants.

### Results

#### Experiment 3a

In Table 1, mean RT and error rates for each experimental condition are shown. Incorrect answers were excluded from the RT analysis (11.97% of the trials), together with anticipations (0.02% of the trials).

##### Irrelevant distractor

**FIGURE 5:**
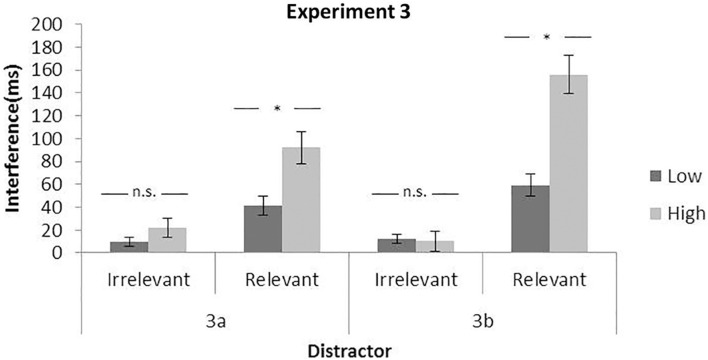
Mean interference effect for irrelevant (distractor present — absent) and relevant (invalid — valid) distractors as a function of perceptual load. Data from Experiment 3. Error bars represent standard error of the mean.

Response time results showed a significant main effect of the load, *F*(1,23) = 231.31, *p* < 0.0001, η*_*p*_*^2^ = 0.91, and the distractor, *F*(1,23) = 10.47, *p* = 0.0037, η*_*p*_*^2^ = 0.31, but no significant interaction between them, *F*(1,23) = 2.08, *p* = 0.1631, η*_*p*_*^2^ = 0.08. In Figure 5, we observe the interference in the high (22 ms) and in the low load condition (9 ms).

The error rate analysis indicated just a significant main effect of the perceptual load, *F*(1,23) = 16.60, *p* = 0.0005, η*_*p*_*^2^ = 0.42. Neither the main effect nor the interaction was significant, both *ps* > 0.1.

##### Relevant distractor

The two main effects of the load and the distractor, as well as their interaction reached significance, *F*(1,23) = 226.07, *p* < 0.0001, η*_*p*_*^2^ = 0.91; *F*(1,23) = 45.84, *p* < 0.0001, η*_*p*_*^2^ = 0.66; and *F*(1,23) = 17.01, *p* = 0.0004, η*_*p*_*^2^ = 0.42, respectively. In this case, as can be observed in Figure 5, the interference was higher in the high (92 ms) than the low load (41 ms) condition.

Error rates presented a significant main effect of the perceptual load and the distractor, *F*(1,23) = 9.25, *p* = 0.0058, η*_*p*_*^2^ = 0.29 and *F*(1,23) = 7.26, *p* = 0.013, η*_*p*_*^2^ = 0.24, respectively, and a significant interaction between them, *F*(1,23) = 13.63, *p* = 0.0012, η*_*p*_*^2^ = 0.37. As can be observed in Table 1, the error rates of the invalid distractor increased on high perceptual load, where participants committed the highest number of errors, while they did not change for the valid distractor.

#### Experiment 3b

Incorrect answers were excluded from the RT analysis (11.59% of the trials), as well as anticipations (0.02% of the trials).

##### Irrelevant distractor

Response times showed a main effect of the load and the distractor, *F*(1,22) = 256.89, *p* < 0.0001, η*_*p*_*^2^ = 0.92 and *F*(1,22) = 5.33, *p* = 0.0308, η*_*p*_*^2^ = 0.19, respectively, but without a significant interaction, *F*(1,22) = 0.05, *p* = 0.8179, η*_*p*_*^2^ = 0.002. In Figure 5, we can observe that there is a small and non-significant difference between the interference in the low (12 ms) and the high load (10 ms) condition.

Error rates showed a significant main effect of the perceptual load, *F*(1,22) = 40.28, *p* < 0.0001, η*_*p*_*^2^ = 0.65, but not of the distractor, *F(*1,22) = 1.68, *p* = 0.2082, η*_*p*_*^2^ = 0.07. However, their interaction was significant, *F*(1,22) = 7.26, *p* = 0.013, η*_*p*_*^2^ = 0.25. Indeed, participants committed more errors on the high than the low load condition, and this difference was higher for the irrelevant distractor than the absent condition (see Table 1).

##### Relevant distractor

The RT analysis showed again significant effects for the load and the distractor, *F*(1,22) = 163.29, *p* < 0.0001, η*_*p*_*^2^ = 0.88 and *F*(1,22) = 101.90, *p* < 0.0001, η*_*p*_*^2^ = 0.82, and the interaction, *F*(1,22) = 32.96, *p* < 0.0001, η*_*p*_*^2^ = 0.60. As can be observed in Figure 5, the interference was higher in the high (156 ms) than the low load (59 ms) condition.

Error rates showed a significant main effect of both the load, *F*(1,22) = 7.04, *p* = 0.0145, η*_*p*_*^2^ = 0.24, and the distractor, *F*(1,22) = 25.57, *p* < 0.0001, η*_*p*_*^2^ = 0.54, as well as a significant interaction, *F*(1,22) = 13.80, *p* = 0.0012, η*_*p*_*^2^ = 0.38. As in Experiment 3a, participants committed more errors with high than low load specially in the invalid condition (see Table 1).

### Discussion

Again, the results are consistent with the previous experiments: (i) in general, RTs and error rates increased in the high compared to the low load condition, and the mere presence of distractors caused interference to the task. (ii) Furthermore, the perceptual load modulated differently the two types of distractor interferences, similar to Experiments 1 and 2. The relevant distractor interference was higher for high load, whereas the irrelevant distractor interference was not again modulated by the load. Importantly, (iii) this consistency in the observed pattern of results points to the type of interference (from potentially relevant vs. fully irrelevant distractors) as an important factor on the modulatory effect of attentional capture.

To further analyze the interaction between perceptual load and the distractor relevance, and to understand the consistent failure to replicate a typical pattern of the results observed in Lavie’s studies, we investigated whether the stimulus features of the irrelevant distractor and, in particular, its dissimilarity with the target, could be part of the explanation (Duncan and Humphreys, 1989; Wolfe, 1994; Bichot and Schall, 1999). In this study, the irrelevant distractors used until now were completely irrelevant to the task in terms of both position and features (dissimilar to the target); this possibly facilitated the withdrawal of attention from them resulting in weak interference. Contrariwise, the relevant distractor was presented on a relevant position (i.e., the red letter within the search array) and also shared features/similarities with the target as being both letters, thus challenging the target/distractor segregation and causing higher interference (Duncan and Humphreys, 1989; Wolfe, 1994; Bichot and Schall, 1999). In the next experiment, we tried to match the similarity of the two distractors—relevant and irrelevant—with the target to investigate the influence of the distractor-target similarity on attentional capture.

## Experiment 4

The task was the same as in Experiments 2a and 3a, except that a non-target letter was used as the peripheral distractor. This experiment series sought to (i) replicate the main previous results while (ii) testing whether the similarity between the target and the irrelevant distractor could increase its interference and consequently now interacts with the perceptual load. (iii) In addition, the sharing of features between the two distractors allows a comparison of them directly with each other.

### Methods

#### Participants

Two new samples participated in the present experimental series, with a total of 25 participants each: Experiment 4a (all right-handed; 15 women; mean age of 25.08 years, SD = 5.22) and Experiment 4b (3 left-handed; 21 women; mean age of 21.25 years, SD = 1.74). One participant was excluded from Experiment 4b due to a low accuracy rate (65%).

#### Procedure and Design

Stimuli and procedure were identical to those of Experiments 2a and 3a, except that the peripheral distractor was a black (Experiment 4a) or red non-target letter (Experiment 4b) instead of an image. The letter was presented at the corresponding position to the center of the image in previous experiments, with the same dimension as the other letters presented in the relevant search array. Furthermore, while typically the distractor letter in the perceptual load studies could be congruent or not with the target, in this case, the identity of the distractor letter was randomly chosen among only the angular letters used as non-target, so that no letter could appear two times simultaneously in the same trial. Doing so, instead of analyzing the response competition (congruent vs. incongruent distractor), we could analyze the effect of the distractor non-target letter as the irrelevant distractor image used until now (the absence vs. presence of the distractor; see Forster, 2013), making the results easier to compare. Again, the experiment consisted of a three-factor design, with all variables manipulated within participants.

### Results

#### Experiment 4a

Again, incorrect trials (12.14%) and anticipations (0.001%) were excluded from the RT analysis. Mean RT and error rates for each experimental condition are shown in Table 1.

##### Irrelevant distractor

**FIGURE 6:**
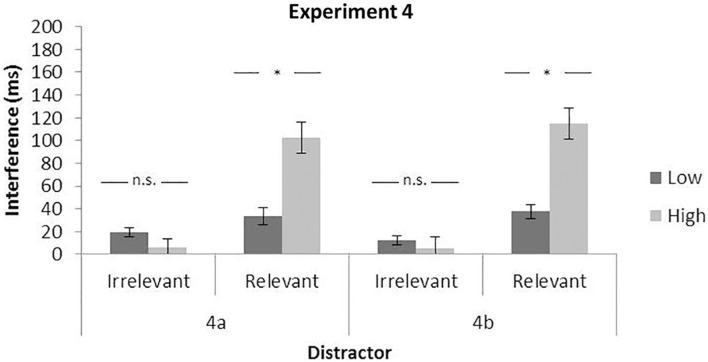
Mean interference effect for irrelevant (distractor present — absent) and relevant (invalid — valid) distractors as a function of perceptual load. Data from Experiment 4. Error bars represent standard error of the mean.

Response time results showed a significant main effect of the perceptual load, *F*(1,24) = 278.77, *p* < 0.0001, η*_*p*_*^2^ = 0.92, and the distractor, *F*(1,24) = 11.01, *p* = 0.0029, η*_*p*_*^2^ = 0.31, but not a significant interaction between them, *F*(1,24) = 2.12, *p* = 0.1579, η*_*p*_*^2^ = 0.08. The interference of the peripheral distractor in the low (19 ms) and in the high load (6 ms) condition is shown in Figure 6.

Error rates showed only a significant main effect of the perceptual load, *F*(1,24) = 25.49, *p* < 0.0001, η*_*p*_*^2^ = 0.51, whereas a main effect of the distractor and an interaction were not significant, both *ps* > 0.1.

##### Relevant distractor

Again, the results showed a main effect of both the perceptual load and distractor, *F*(1,24) = 235.85, *p* < 0.0001, η*_*p*_*^2^ = 0.91 and *F*(1,24) = 64.92, *p* < 0.0001, η*_*p*_*^2^ = 0.73, together with a significant interaction, *F*(1,24) = 23.57, *p* < 0.0001, η*_*p*_*^2^ = 0.49. As can be observed in Figure 6, the interference was higher with high (102 ms) than low perceptual load (34 ms). The analysis of the error rates presented only the two significant main effects of the perceptual load and the distractor, *F*(1,24) = 9.38, *p* = 0.0053, η*_*p*_*^2^ = 0.28 and *F*(1,24) = 10.42, *p* = 0.0036, η*_*p*_*^2^ = 0.30. However, the interaction failed to reach significance, *F* (1,24) = 1.07, *p* = 0.3115, η*_*p*_*^2^ = 0.04.

#### Experiment 4b

Incorrect responses (13.12% of the trials) and anticipations (0.04% of the trials) were excluded from the RT analysis.

##### Irrelevant distractor

Response time results showed a main effect only of the load, *F*(1,23) = 345.52, *p* < 0.0001, η*_*p*_*^2^ = 0.94, while a main effect of the distractor and its interaction with the load failed to reach significance, respectively, *F*(1,23) = 2.31, *p* = 0.142, η*_*p*_*^2^ = 0.09, and *F*(1,23) = 0.52 *p* = 0.479, η*_*p*_*^2^ = 0.02. The interference of the peripheral distractor in low (12 ms) and high load conditions (5 ms) can be observed in Figure 6.

Error rates presented a significant main effect only for the perceptual load, *F*(1,23) = 18.12, *p* = 0.0003, η*_*p*_*^2^ = 0.44. The distractor and its interaction with the load failed to reach significance, both *Fs* < 1.

##### Relevant distractor

Response times showed a main effect of both the perceptual load and distractor, *F*(1,23) = 469.51, *p* < 0.0001, η*_*p*_*^2^ = 0.95 and *F*(1,23) = 73.76, *p* < 0.0001, η*_*p*_*^2^ = 0.76, and a significant interaction, *F*(1,23) = 37.52, *p* < 0.0001, η*_*p*_*^2^ = 0.62. Again, as can be observed in Figure 5, the interference was higher on the high (115 ms) than the low load condition (38 ms).

Error rates showed only significant main effects of the load and distractor, *F*(1,23) = 11.47, *p* = 0.003, η*_*p*_*^2^ = 0.33 and *F*(1,23) = 19.52, *p* = 0.0002, η*_*p*_*^2^ = 0.46, but not for the interaction, *F*(1,23) = 4.25, *p* = 0.051, η*_*p*_*^2^ = 0.16.

### Discussion

Overall, the results show a high consistency with the previous experiments: (i) the perceptual load manipulation was always significant for both RTs and error rates, with an increment in the high compared to the low perceptual load condition as also the mere presence of distractors, in general, caused interference to the task. (ii) In addition, potentially relevant and irrelevant distractors differently interacted with the load even though, especially in Experiment 4b (peripheral red letter), they were identical in terms of features. Indeed, the relevant distractor interference clearly increased with a high load while, once again, the irrelevant distractor did not interact with the load. To conclude, (iii) the fact that the interference produced by characteristically equal distractors, within the same experimental procedure and task, was qualitatively different (i.e., modulated or not by perceptual load) supports the idea that the relevance of the distractor, and therefore its relationship with the target, is an important variable explaining the opposite effects previously observed in Lavie’s and Gaspelin’s attentional capture studies.

However, it is important to emphasize that we have based our analysis on the definition of the interference used by the two original theories, the perceptual load, and the attentional dwelling, so that when comparing these theories, we must take into account how attentional capture is defined by each. Indeed, while in the perceptual load theory the interference of the (irrelevant) distractor is defined as the difference between the presence and absence of the distractor, in the attentional dwelling hypothesis the (relevant) distractor interference is considered as the difference between the invalid and valid distractor. Thereby, while the interference of the irrelevant distractor represents merely the cost of its presence, this is not the case for the relevant distractor interference. Indeed, calculating attentional capture as a validity effect prevents us from determining whether the observed interference depends on the cost of the distractor’s presence (i.e., invalid distractor non-target letter colored in red) vs. its absence (no letter colored in red) and/or by the benefits of presenting a salient target (i.e., the valid distractor or target colored in red).

For this reason, we decided to analyze collectively the results of all the experiments in this study, comparing each distractor condition with the absent condition. By doing so, we would have three comparable indexes: (i) the interference from the peripheral fully irrelevant distractor (absent vs. peripheral distractor) is calculated as it has been done until now for the irrelevant distractor; (ii) the interference from the invalid potentially relevant distractor (absent vs. invalid search display distractor), which represents the actual cost of the presence of the potentially relevant distractor, and (iii) attentional capture by the valid distractor, i.e., by the target (absent vs. valid), indicating the benefit of presenting a salient target. In this way, this analysis allows us to separate the interference produced by the relevant distractors in the two different indexes—the cost of the invalid red letter and the benefit of the target red letter—and to understand the weight of each on the results of this study (the validity effect). This will allow a fairer comparison between the modulation of perceptual load over the interference produced by fully irrelevant and potentially relevant distractors.

## Combined Overall Analysis

For this statistical analysis, a linear mixed-effects model was conducted in R (R Core Team, 2020), with the lme4 (Bates et al., 2015), lmerTest (Kuznetsova et al., 2017), and phia (De Rosario-Martinez, 2015) R packages, version 4.0. The linear mixed-effects model allows estimating and simultaneously control for the variance and covariance components of random effects caused by the variability between participants and experiments.^2^ Because we were still interested in the interaction between the perceptual load and distractor interferences, we used RTs of all the observations as a dependent variable (no. of observations = 71,600), so that we had enough trials to observe the interaction, with the perceptual load (low and high) and the distractor conditions (absent, peripheral, valid, and invalid) as the fixed effects of the model. Regarding the random effects, we took into account the participants and the experiments. For the latter, we considered each experiment of the series as a variable of this condition, resulting in a total of seven levels (Experiments 1, 2a, 2b, 3a, 3b, 4a, and 4b). To find a random structure, we first determined its maximal converging model, and then the best fitting model was chosen using the restricted maximum likelihood (REML), so that the random effects of the final model considered the random intercept for experiment and the slope of the load for participants.

The analysis revealed that the best model included a significant main effect of both the distractor condition, *F*(3) = 492.27, *p* < 0.0001, and the load, *F*(1) = 2,088.52, *p* < 0.0001, as well as a significant interaction, *F*(3) = 136.32, *p* < 0.0001. Finally, to observe the three indexes of interest, we created three subsets, each one with just the absent condition and one of the other distractor conditions (i.e., peripheral, valid, or invalid), and analyzed them with the test Interactions function from the phia package adjusted by Bonferroni.

The results of the peripheral/irrelevant distractor showed significant interference effects for both the low (14 ms), χ^2^(1) = 34.46, *p* < 0.0001, and high perceptual load (13 ms), χ^2^(1) = 26.11, *p* < 0.0001; importantly, however, this type of interference did not interact with the perceptual load, χ^2^(1) = 0.06, *p* = 0.8051, with less than 1 ms of difference between the two conditions. Interestingly, and in contrast to the peripheral/irrelevant distractor interference, the interference from the potentially relevant distractor, i.e., the cost of the invalid distractor, interacted significantly with the load, χ^2^(1) = 9.90, *p* = 0.0016, with larger interference for high load. This modulation of the load for the latter was significantly different from the lack of modulation observed for the former, χ^2^(1) = 6.67, *p* = 0.0098.

**FIGURE 7:**
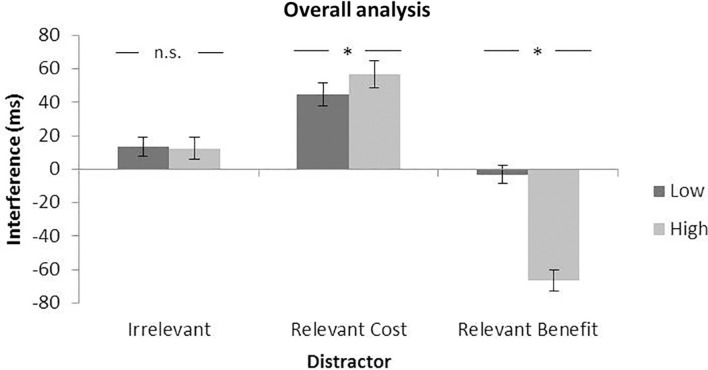
Mean interference effect for irrelevant, relevant cost, and relevant benefit compared to the absent condition (crossing of the axis). Data Combined from all the experiments of this study. Error bars represent standard error of the mean.

In addition to our crucial comparison of the two interference effects, the modulation of the load over the benefits observed for the valid condition was clearly significant, χ^2^(1) = 374.11, *p* < 0.0001; in fact, we observed (see Figure 7) a much larger modulation for the benefits (64 ms larger for high compared to low perceptual load), than for the costs (11 ms larger for high perceptual load).

In sum, while the lack of modulation of the perceptual load over the interference produced by a fully irrelevant peripheral distractor, consistently observed over the seven experiments, remains constant, the same produced within the search array by the potentially relevant distractor was significantly modulated by load, with costs (and benefits) increasing with a high perceptual load. On one hand, this last analysis confirms the failure to find the perceptual load modulation over the peripheral distraction typically observed by Forster and Lavie (2008a, 2016) and Morris et al. (2020). On the other hand, even when the definition of the interference from the potentially relevant distractor was mirrored to the definition of the interference from the fully irrelevant distractor (i.e., the cost of presenting the distractor within the search array), the observed pattern of the results seems to be in line with the attentional dwelling hypothesis (larger interference with high load) rather than with Lavie’s perceptual load theory.

However, it is interesting to note that the index mostly affected by the load seems to be the valid distractor, indicating the possibility that the pattern of the validity effect used in the attentional dwelling hypothesis could depend more on the benefit of having a salient target than the cost of presenting a salient non-target stimulus.

## General Discussion

To summarize, with the present experimental series, we tested the two main theories, the perceptual load and the attentional dwelling hypothesis, within the same paradigm, manipulating both perceptual load, low or high, and the distractor relevance, potentially relevant vs. fully irrelevant. Seven experiments were conducted to investigate why research supporting the two theories typically observe opposite effects, with the hypothesis that the distractor relevance would be a critical aspect in explaining the discrepancies between the abovementioned theories. We argue that the mechanisms underlying the processing of different distractor types (relevant and irrelevant) are different; therefore, also their interference to the task and their interaction with the load will differ. In particular, we expected that when the perceptual load of the task increases from low to high, irrelevant distractors would elicit weaker interference, as predicted by the perceptual load theory, while relevant distractors would trigger a stronger effect under high load, as predicted by the attentional dwelling hypothesis.

Interestingly, all the experiments presented in this study showed similar results, independently of different task variations, which can be summarized in three main findings: (1) In contrast to most previous research conducted under the umbrella of Lavie’s perceptual load theory (but in line with some other studies; e.g., Biggs et al., 2012; Lleras et al., 2017) perceptual load did not modulate the interference from entirely task-irrelevant distractors. (2) In contrast, the expected modulation of the interference by the potentially relevant distractor was consistently observed throughout the seven experiments, with a larger effect under high load. And interestingly, for the first time to our knowledge, we disentangled that high perceptual load increased both the benefits of making salient the target and the interfering cost of making salient one of the distractors. (3) Even when using a fairer comparison between the interference by irrelevant distractors (from outside the attentional focus) and that from potentially relevant distractors within the attentional focus, the two types of interference seem to be qualitatively modulated differently by the perceptual load/search difficulty. These important findings are discussed in the following in this order.

Despite the presence of all the manipulations, the interference of the fully irrelevant distractor always failed to interact with the load, causing the same (small) interference in both low and high perceptual load. To the best of our knowledge, a few studies have reported similar results, with the distractor interference also in high load, and most of them have used mostly faces or familiar objects as distractors (Lavie et al., 2003; Schall et al., 2003; Ro et al., 2009; Neumann et al., 2011; Biggs et al., 2012). This phenomenon led Lavie (2005) to explain the use of faces as distractors as a special case, in which stimuli with a high degree of familiarity/expertise (e.g., a musical instrument for musicians or logos and flags) and especially faces, continue to interfere also in high load (Forster, 2013; Thoma and Lavie, 2013; Murphy et al., 2016). However, familiarity does not seem to explain the pattern of our results, and also other studies that tried to give an alternative explanation to the perceptual load results did find different outcomes. In particular, the well-known dilution theory (Benoni and Tsal, 2010, 2013) argues that the interference of the distractor is diluted by all the stimuli presented in the array, even if the distractor processing is equivalent in both load conditions. Indeed, according to this theory, the typical perceptual load results depend on dilution rather than on the perceptual load. While in a low perceptual load task, the target usually pops out from the non-target letters, and they could be considered as the same stimulus because of their homogeneity, in a high load task the presentation of multiple non-target letters, dilutes the interference of the distractor with them. In their experiments, Benoni and Tsal (2010, 2013) manipulated both load and dilution, showing that the distractor still interferes in high load when dilution is low. Considering this theory, the presentation of the letters inside the two separated boxes could be adding more stimulus in which attention is diluted. So that, compared to a typical perceptual load paradigm in which all letters are presented in a unique circular array, the division of these stimuli in two boxes could extend the number of stimuli in which attention is diluted.

Another interesting explanation was made by Biggs and Gibson (2018), in which they studied the attentional window hypothesis (Belopolsky et al., 2007; Belopolsky and Theeuwes, 2010) with the perceptual load paradigm. According to this hypothesis (Belopolsky et al., 2007), the distractor processing within or outside the attentional window depends on different mechanisms, while the size of the attentional window is controlled by top-down mechanisms, this control is not possible with stimuli within the attentional window, where attention is guided by bottom-up processes. Furthermore, aspects like task difficulty seemed to determine the size of the window, the more difficult the search the smaller the size, explaining the reduced interference of stimulus outside the attentional window like with irrelevant distractors (Theeuwes, 2010). In a series of experiments, Biggs and Gibson (2018) observed that when the irrelevant distractor is not clearly separated by the relevant search array, its presence interferes also in high load conditions. They concluded that attentional capture is dominated by the attentional window rather than the perceptual load and that a clear definition of the relevant search array (e.g., circular array or boxes) could be preventing the attentional capture of the irrelevant distractor.

An alternative explanation was proposed by Kerzel et al. (2012), who presented two arrays at the same time, and observed that the distractor caused task interference only when shown in the target array. The authors concluded that all the stimuli presented in the target array were perceptually grouped, thus preventing attentional capture from other stimuli. Under this view, the lack of interaction between the irrelevant distractor and the perceptual load could rely on the two boxes, which delimitate the relevant search array from the irrelevant distractor even more than a circular search array, causing a separation/grouping effect.

Even if the paradigm used here presents small details that differ from the original perceptual load paradigm such as stimuli time exposure, background color, and/or letters position among others, which could be preventing its replication, based on both the aforementioned theories, it also seems that using boxes may have an important influence on the interference of the irrelevant distractor. Indeed, compared to other studies (Forster and Lavie, 2008b; Lleras et al., 2017; Morris et al., 2020), the interference of the irrelevant distractor is much smaller (∼13 ms) than what is typically observed (∼50 ms, Forster and Lavie, 2008b); although it is important to note that in almost all of our experiments (except Experiments 2a and 4b), a main effect of the irrelevant distractor was significant. Another possible explanation about this small but significant interference by the irrelevant distractor could be understood as a reflection of a filtering cost (Folk and Remington, 1998), wherein the distractor still interferes with the task even if it does not actually capture the attention of the observer.

At any rate, even accepting that these small differences from the original perceptual load paradigm could be diminishing the interference of the distractor, this systematic failure, across the seven experiments, to replicate previous classical results with the perceptual load paradigm is in line with a recent study by Lleras et al. (2017), wherein the authors were also unable to replicate the findings of Forster and Lavie (2008b) when presenting small changes from the original paradigm such as the proportion of target present trials, the type of irrelevant distractor, and the display duration. Interestingly, they were able to replicate the classical perceptual load results of the irrelevant distractor but only and exclusively when using the very same paradigm used by Forster and Lavie (Lleras et al., 2017).

Therefore, further research is necessary to investigate why, although the modulation of perceptual load over capture by completely irrelevant distractors can be replicated when exactly the same set of original parameters is used, such a modulation disappears with slight procedural modifications. In any case, the abovementioned failures to replicate, together with ours, suggest at least that a typical perceptual load modulation over fully irrelevant distractors does not easily generalize to procedures where small changes in *a priori* non-critical variables are introduced, conjointly with the manipulation of perceptual load. More studies are needed to better understand the weight of each of these variables as the modulators of these effects.

On the other hand, the observed results of the potentially relevant distractor interference are consistent with the attentional dwelling hypothesis even without using abrupt onset cues. These findings, together with similar results (Barras and Kerzel, 2017), suggest that the main effects associated with this theory can be easily replicable and generalized (Gaspelin et al., 2016; Ruthruff et al., 2020). Indeed, Barras and Kerzel (2017) found a greater interference of a color singleton distractor when the search tasks were difficult, similarly to our results, but using a typical additional singleton paradigm. However, contrary to an additional singleton paradigm, in which the interference represents the cost of presenting the distractor compared to its absence, the validity effect typically used to measure attentional capture in this framework does not differentiate between the cost of the invalid distractor and the benefit of the valid distractor. The presence of a condition without any distractor allowed us to separate these interferences, showing that the observations made by the attentional dwelling hypothesis seem to depend more on the benefits of presenting the valid distractor than on the attentional costs elicited by the invalid distractor. Interestingly, these results do not seem to contradict completely the predictions made by the attentional dwelling hypothesis. Indeed, in a recent study, Ruthruff et al. (2020) affirm that according to this hypothesis both costs and benefits of capture depend on the task search difficulty, more exactly on “how long spatial attention dwells on the non-target items during visual search” (Ruthruff et al., 2020, p.1). This way, attentional capture would be latent on an easy search and visible only in difficult search tasks, for both valid and invalid distractors.

These results are also supported by the priority accumulation framework (Lamy et al., 2018; Gabbay et al., 2019), which claims that in each location of the array, there is an accumulation of attentional priority over time, which eventually will turn on the selection of the highest priority location so that a cue validity effect represents the duration of the target-distractor competition. In this context, a valid cue accelerates the resolution of the competition. Specifically, the authors argue that the target selection is faster when the target has the highest priority (e.g., valid red letter), and that the target-distractor competition will be easy to solve in an easy search trial (i.e., when target-distractor similarity is low) than in a difficult search (i.e., when target-distractor similarity is high) because in the first case the competition is easier to solve compared to the second one (Darnell and Lamy, 2020). Therefore, valid cues will facilitate the target selection especially with high task difficulty (i.e., when there is a large target-distractor competition). This could explain the observed interaction between the valid distractor and the load. Greater difficulty opens up the window for a greater benefit with the presence of a valid distractor.

Finally, and perhaps more importantly, we suggest that the relevance of the distractor (from inside vs. outside the attentional focus) is a crucial aspect when interpreting these results as the different interactions with the load/search difficulty may reflect the different mechanisms involved in the regulation of these sources of interference. Interestingly, some perceptual load studies have demonstrated that when the same distractor stimulus is presented peripheral to the target array or at the fixation position, the interference was similarly modulated as in the typical perceptual load studies, independently of its position (Beck and Lavie, 2005). However, other studies found that high perceptual load tends to increase the distractor interference when this forms a part of the target (e.g., Stroop task) (Chen, 2003; Cosman and Vecera, 2012), indicating that, even if we do not observe any interaction of the irrelevant distractor with the load, the results are partially in line with other perceptual load studies.

This evidence, along with our study, may indicate that depending on the relevance of the distractor to the target dimension, different mechanisms are involved in controlling the distractor interference.

More specifically, a possible explanation could be that participants first define the attentional set, that is, which stimuli are potentially relevant and which are fully irrelevant, and only afterward select the target among other potentially relevant stimuli. On one hand, the attentional set can be established in advance at the beginning of the experiment or learned across trials, but definitely, before a target display is presented in a given trial so that we can voluntarily focus our attention and resources on the preselected area or dimension/s. On the other hand, the target selection is a trial-by-trial process that depends on the target-distractor competition and cannot be established in advance. This idea could be also supported in terms of spatial filtering (Wang and Theeuwes, 2018) as it seems that those locations where distractor stimuli are often presented tend to be suppressed, so that stimuli in those locations, like the irrelevant distractor, are easily ignored, resulting in weaker attentional capture (Ruthruff and Gaspelin, 2018). Thus, while the items at relevant locations cannot easily be ignored and are under a perceptual load effect, irrelevant distractors can readily be ignored by spatial filtering, consequently not being modulated by the perceptual load. According to our view, the selection of the relevant area or dimension would be proactively guided mainly by explicit goals/top-down mechanisms in the short term and by learning mechanisms in the long term (Luck et al., 2021); whereas actual target selection rather depends on the trial-by-trial competition between top-down and bottom-up factors. Consequently, also the mechanisms underlying attentional capture should be different depending on the nature of the distractor (fully irrelevant or potentially relevant).

In neural terms, these different mechanisms could be accommodated within the attentional network model by Corbetta and Shulman (2002), and be relevant to the discussion about whether exogenous attention is implemented on the ventral frontoparietal network (Chica et al., 2011) or both exogenous and endogenous attention mechanisms are implemented in the dorsal frontoparietal network (Corbetta et al., 2008). In the case of fully or highly irrelevant distractors, attentional capture may rely on a balance between the dorsal and ventral attentional network, which works in an anticorrelated way. Therefore, even if the ventral network is considered as a “circuit breaker” of the ongoing task when a novel but irrelevant stimulus is presented, its response may be somehow filtered by the proactive inhibition through the deactivation of the dorsal network on the basis of expectancies (Kincade et al., 2005; Corbetta et al., 2008; Doricchi et al., 2010), and indexed by a reduced P1 on invalid trials (Lasaponara et al., 2011). It is possible that an obvious separation between the target and fully irrelevant distractors could make this control easier, opening the door to proactive inhibition or habituation. However, when the distractor could potentially be the target, its selection would be the result of a competition attentively mediated exclusively by the dorsal network and the involvement of stimulus-driven and top-down control mechanisms. This potentiality of being the target, in this case, would eliminate the possibility of habituation and proactive inhibition, thus allowing only a more reactive inhibition of the distractor.

Finally, the proliferation of studies and theories on attentional capture has made the need to delineate several aspects of this process even more evident. On one hand, many theories like the perceptual load theory need to be renewed to embrace the new findings and questions of the field. On the other hand, this study also highlights the need for a better definition of attentional capture and how to study it, raising questions about whether the validity effect, the distractor presence cost, or the response competition all could represent attentional capture or maybe just different aspects of the same effect.

To conclude, we consider that the results obtained from this experimental series could have an important impact on future conceptualizations and definitions of attentional capture. Indeed, conjointly studying the attentional dwelling and the perceptual load theory for the first time, it shows that mixed results obtained from both original theories did not depend on the type of paradigm used, rather by different processes underlying attentional capture by each distractor type. Moreover, future use of this paradigm could help to disentangle different top-down mechanisms (both explicit and implicit) and their interaction with bottom-up processing during selective attention. In this regard, it is worth noting that, even if the relevant distractor used here can be defined as a singleton distractor, its differences from that used in an additional singleton paradigm (i.e., color distractor on the target location) and the contingent capture paradigm (i.e., the probability of the distractor of being the target), may preclude the possibility of drawing general conclusions about the attentional capture debate. However, Barras and Kerzel (2017) used an additional singleton paradigm with the search difficulty manipulation and observed similar results to the one presented in this study (i.e., larger interference in high search difficulty), an aspect that supports the idea that further development of this perceptual load/search difficulty paradigm can provide new insights into this debate as well. In particular, while Gaspelin et al. (2016) conclude that attentional capture studies should mainly use difficult visual search tasks to detect and study a capture effect (Gaspelin et al., 2016; Ruthruff et al., 2020), we also suggest that we could understand more about capture effects through its interaction with the load/search difficulty. Indeed, even if this study suggests that perceptual load is not a core factor in explaining attentional capture, it is certainly a relevant one. The observation of how attentional capture interacts with task demands could give us more insights than simply observing it in one type of task.

## Data Availability Statement

All data are available online at https://osf.io/r27yp/, and Experiment 3 was preregistered prior to data collection (https://osf.io/neujm). Data from Experiments 1 and 2a are available in this academic repository: https://www.repository.unipr.it/.

## Ethics Statement

The studies involving human participants were reviewed and approved by University of Granada committee with the code 175/CEIH/2017. The patients/participants provided their written informed consent to participate in this study.

## Author Contributions

GM collected the data for Experiments 1 and 2a under the supervision of VF. EF collected the data for Experiment 2b under the supervision of JL and EM-A. MF collected the data for Experiment 4a under the supervision of JL, FB, and EM-A. All authors contributed to the article and approved the submitted version.

## Conflict of Interest

The authors declare that the research was conducted in the absence of any commercial or financial relationships that could be construed as a potential conflict of interest.

## Publisher’s Note

All claims expressed in this article are solely those of the authors and do not necessarily represent those of their affiliated organizations, or those of the publisher, the editors and the reviewers. Any product that may be evaluated in this article, or claim that may be made by its manufacturer, is not guaranteed or endorsed by the publisher.

## Footnotes

1 Note that similar natural/neutral images have shown to be effective distractors in previous paradigms (Martín-Arévalo et al., 2015; de Haro et al., 2020).

2 Note that we also conducted an ANOVA combining all the data from this study, observing similar results to the linear-mixed effect model.
